# Supplementary material for: Metformin protects trabecular meshwork against oxidative injury via activating integrin/ROCK signals
Source: eLife. 2023 Jan 4;12:e81198. doi: 10.7554/eLife.81198 (PMC9812404; doi:10.7554/eLife.81198)

Figure 5-source data2 integrin


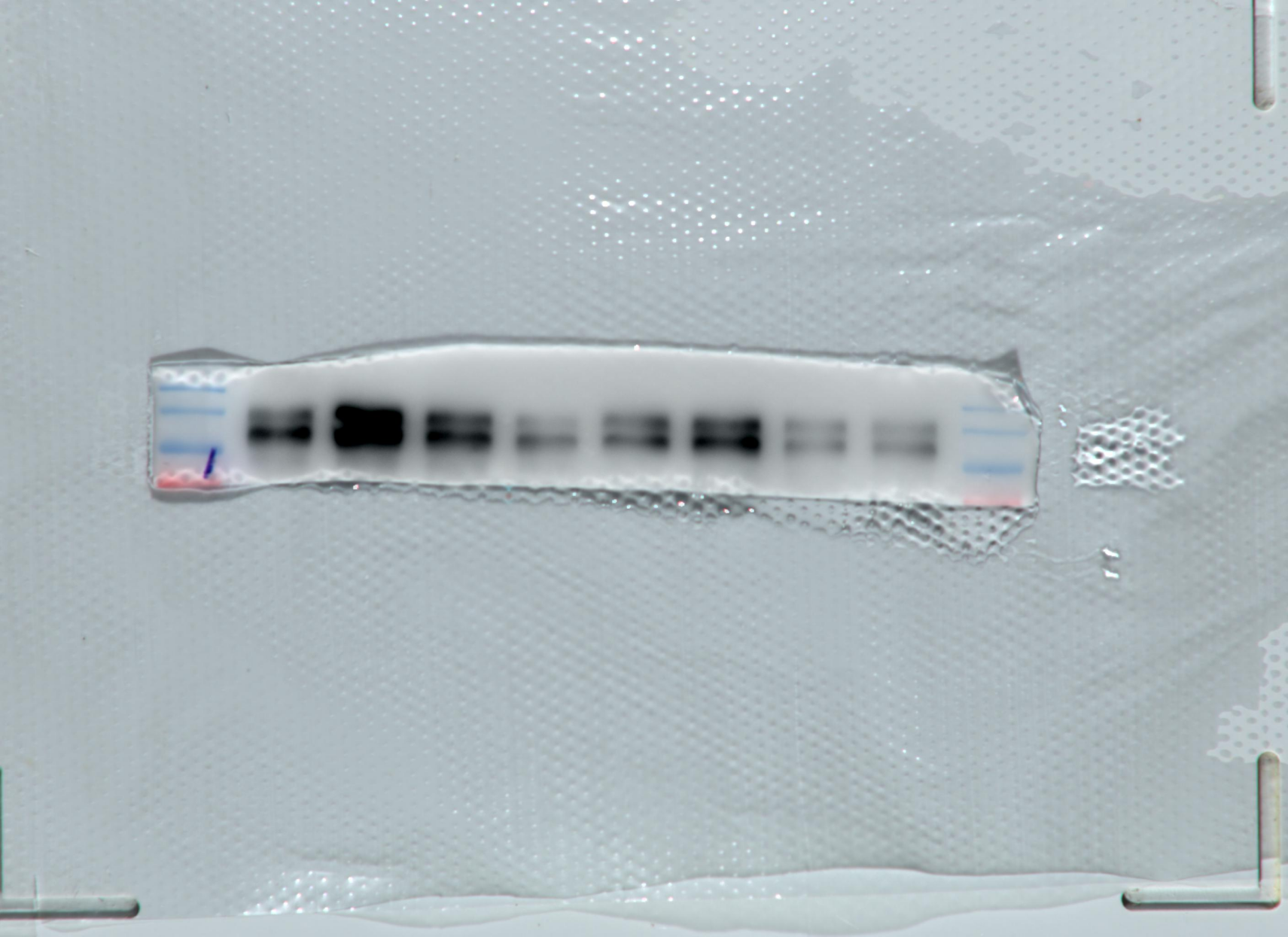


Figure 5-source data3 ROCK 1/2


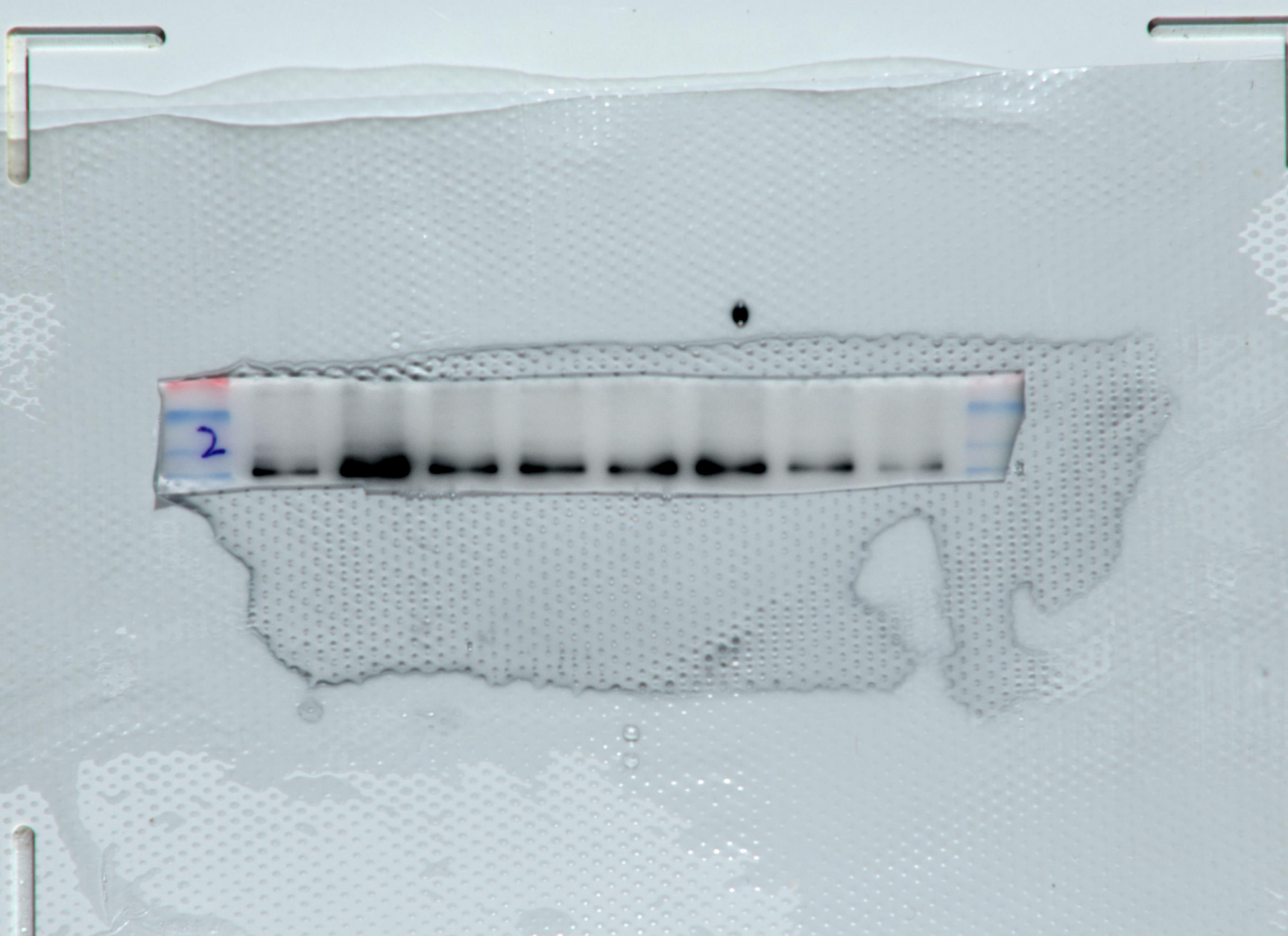


Figure 5-source data4 AMPK


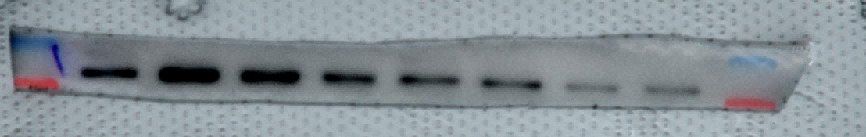


Figure 5-source data5 pAMPK


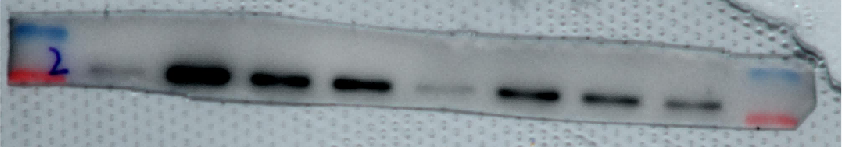


Figure 5-source data6 MLC 1


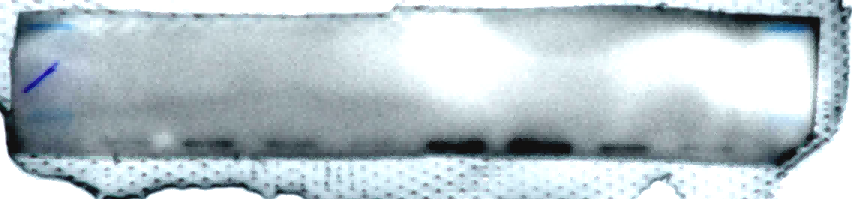


Figure 5-source data7 F-actin


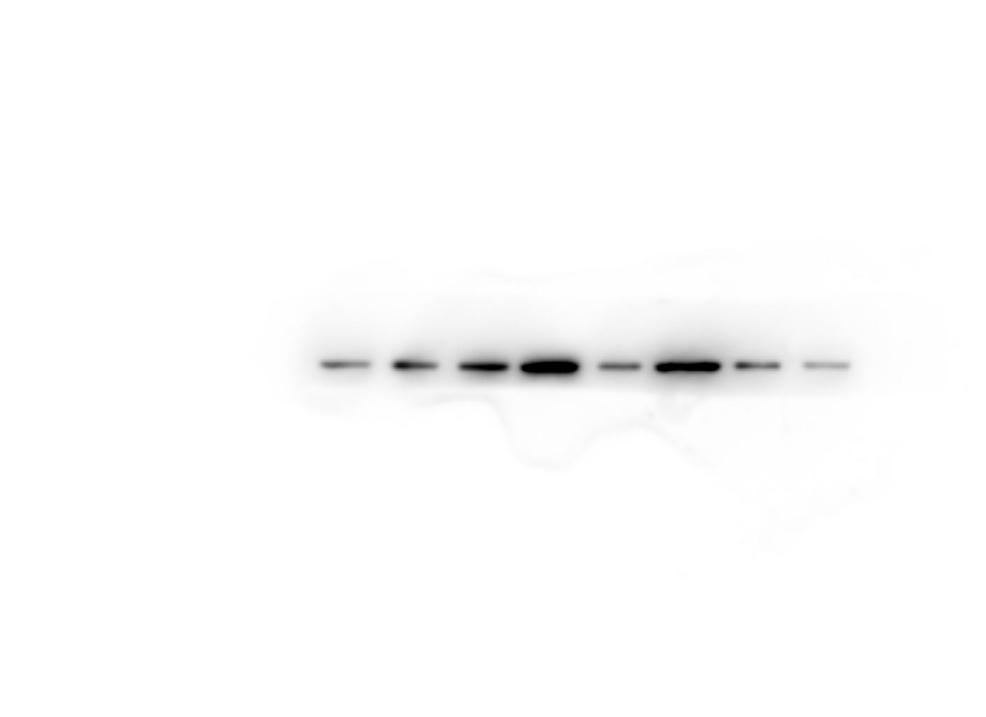


Figure 5-source data8 β-actin


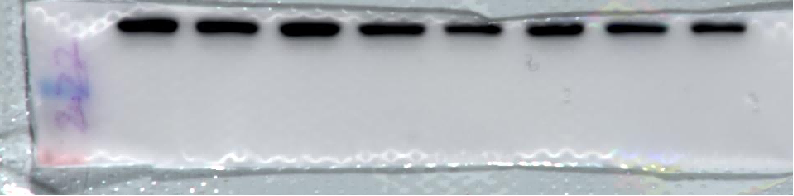

Supplement: Source data 1. [file elife-81198-data1.zip › MET-souce data 20221103/Figure5-source data9.docx]
