# Supplementary material for: Metformin protects trabecular meshwork against oxidative injury via activating integrin/ROCK signals
Source: eLife. 2023 Jan 4;12:e81198. doi: 10.7554/eLife.81198 (PMC9812404; doi:10.7554/eLife.81198)

Figure 5-source data2   integrin

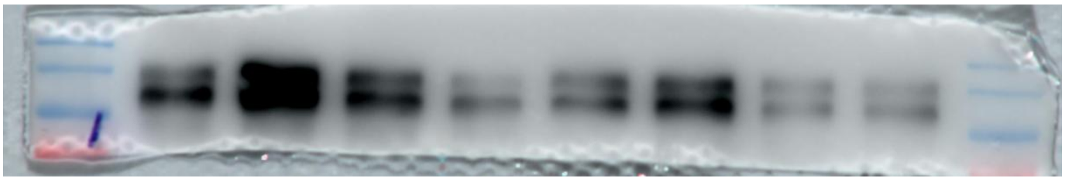

Figure 5-source data3   ROCK 1/2

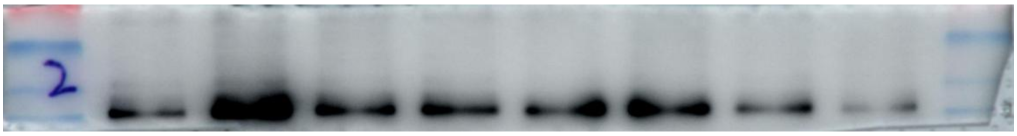

Figure 5-source data4   AMPK

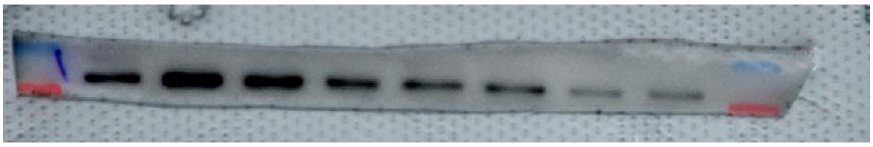

Figure 5-source data5   pAMPK

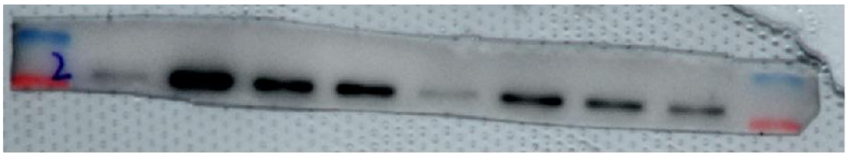

Figure 5-source data6   MLC 1

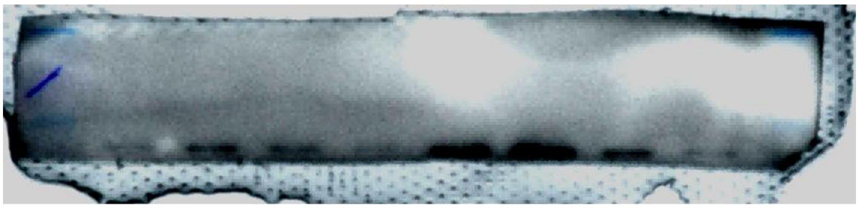

Figure 5-source data7   F-actin

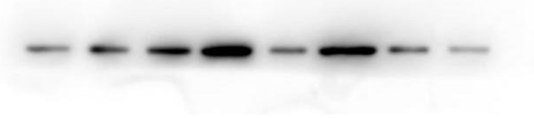

Figure 5-source data8 β-actin

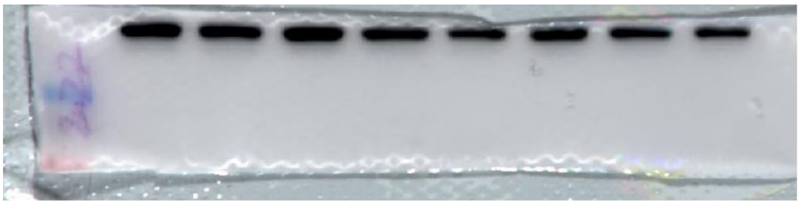

Supplement: Source data 1. [file elife-81198-data1.zip › MET-souce data 20221103/Figure5-source data9.pdf]
