# Supplementary figures and images for: Metformin protects trabecular meshwork against oxidative injury via activating integrin/ROCK signals
Source: eLife. 2023 Jan 4;12:e81198. doi: 10.7554/eLife.81198 (PMC9812404; doi:10.7554/eLife.81198)

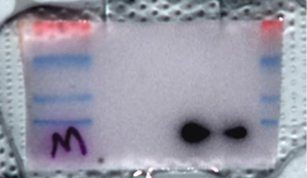

Supplement: Source data 1. [file elife-81198-data1.zip › MET-souce data 20221103/Figure 4-source data3(myocilin).png]

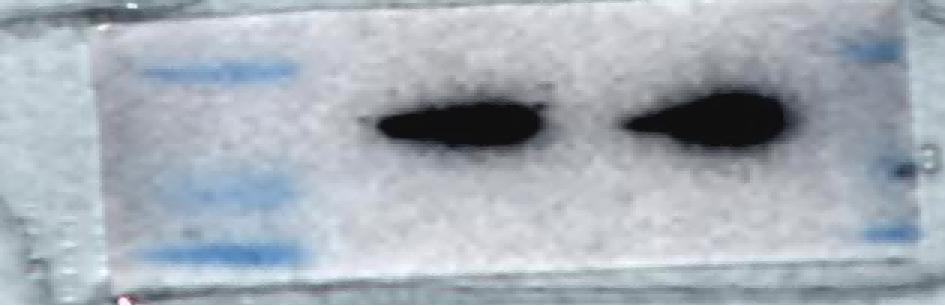

Supplement: Source data 1. [file elife-81198-data1.zip › MET-souce data 20221103/Figure 4-source data4(a┬-actin).png]

Figure 4-source data3 myocilin


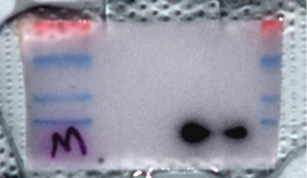


Figure 4-source data4 β-actin


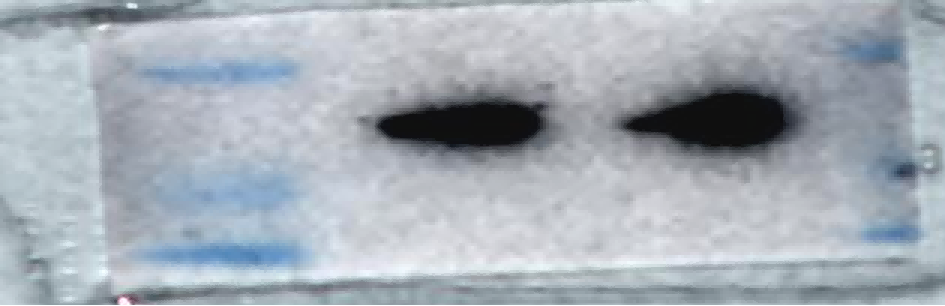

Supplement: Source data 1. [file elife-81198-data1.zip › MET-souce data 20221103/Figure4-source data5.docx]

Figure 4-source data3 myocilin

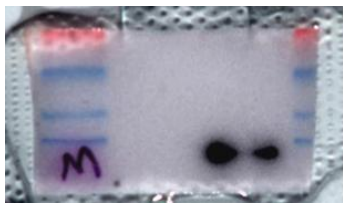

Figure 4-source data4  $\beta$ -actin

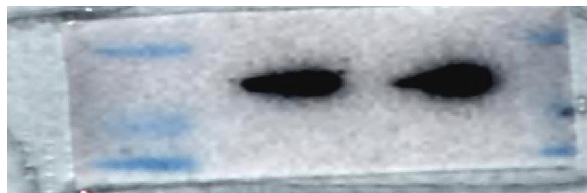

Supplement: Source data 1. [file elife-81198-data1.zip › MET-souce data 20221103/Figure4-source data5.pdf]

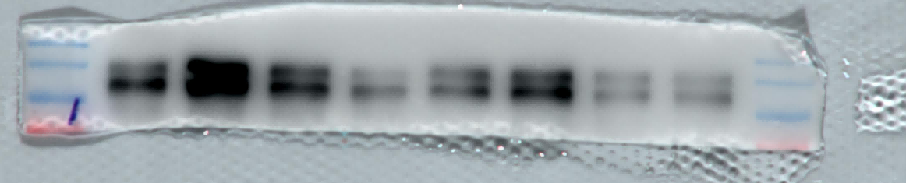

Supplement: Source data 1. [file elife-81198-data1.zip › MET-souce data 20221103/Figure5-source data2 (integrin).png]

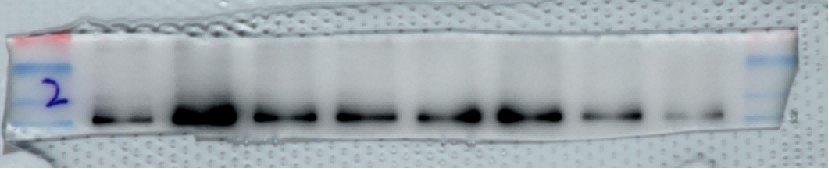

Supplement: Source data 1. [file elife-81198-data1.zip › MET-souce data 20221103/Figure5-source data3 (ROCK).png]

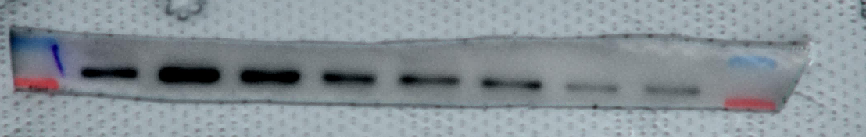

Supplement: Source data 1. [file elife-81198-data1.zip › MET-souce data 20221103/Figure5-source data4 (AMPK).png]

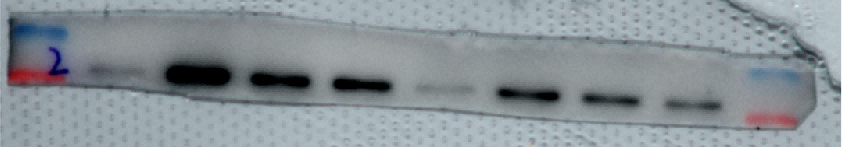

Supplement: Source data 1. [file elife-81198-data1.zip › MET-souce data 20221103/Figure5-source data5 (pAMPK).png]

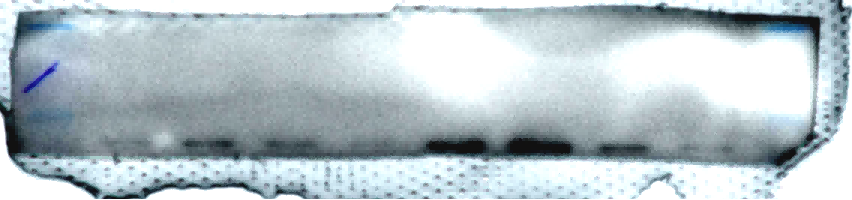

Supplement: Source data 1. [file elife-81198-data1.zip › MET-souce data 20221103/Figure5-source data6 (MLC1).png]

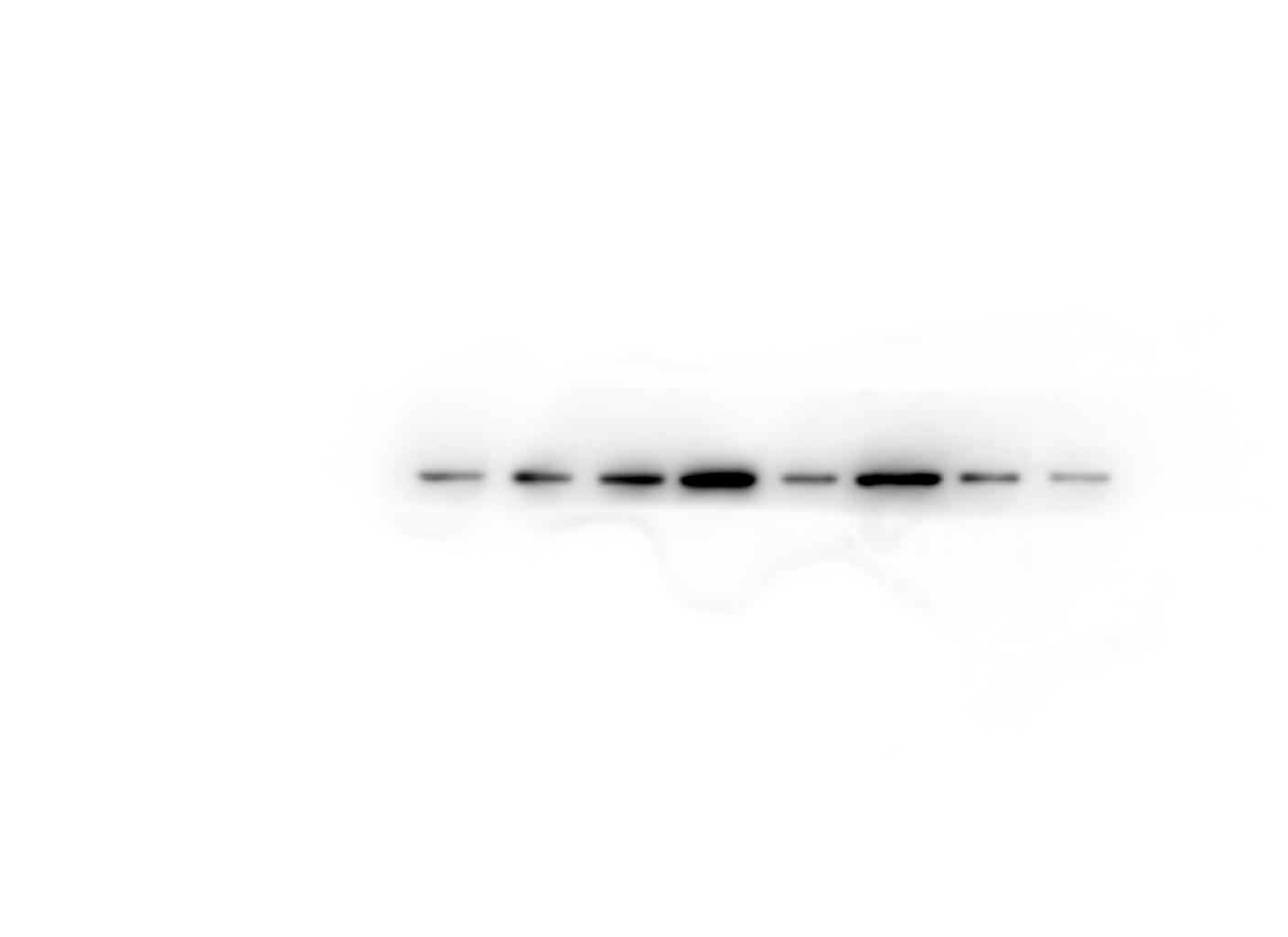

Supplement: Source data 1. [file elife-81198-data1.zip › MET-souce data 20221103/Figure5-source data7 (F-actin).jpg]

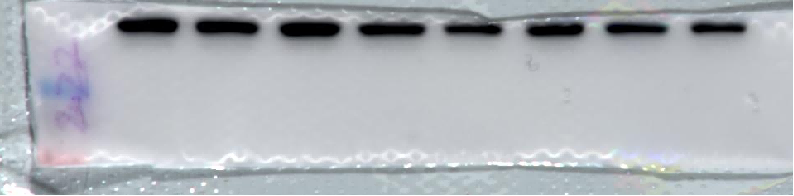

Supplement: Source data 1. [file elife-81198-data1.zip › MET-souce data 20221103/Figure5-source data8 (a┬-actin).png]
